# Correction: Differential recordings of local field potential: A genuine tool to quantify functional connectivity

**DOI:** 10.1371/journal.pone.0228147

**Published:** 2020-01-16

**Authors:** Gabriel Meyer, Julien Carponcy, Paul Antoine Salin, Jean-Christophe Comte

## Notice of Republication

This article was republished on January 9, 2020, to correct errors in the author byline and citation affecting all author names. Please download this article again to view the correct version. The originally published, uncorrected article and the republished, corrected articles are provided here for reference.

## Supporting information

S1 File Originally published, uncorrected article. (PDF)

S2 File Republished, corrected article. (PDF)
